# Deep Brain Stimulation beyond the Clinic: Navigating the Future of Parkinson’s and Alzheimer’s Disease Therapy

**DOI:** 10.3390/cells12111478

**Published:** 2023-05-25

**Authors:** Degiri Kalana Lasanga Senevirathne, Anns Mahboob, Kevin Zhai, Pradipta Paul, Alexandra Kammen, Darrin Jason Lee, Mohammad S. Yousef, Ali Chaari

**Affiliations:** 1 Weill Cornell Medicine-Qatar, Education City, Qatar Foundation, Doha 24144, Qatar; 2 Department of Neurosurgery, Keck School of Medicine, University of Southern California, Los Angeles, CA 90033, USA; 3 USC Neurorestoration Center, University of Southern California, Los Angeles, CA 90033, USA

**Keywords:** neurosurgery, neurology, geriatrics, dementia, dyskinesia, aging, deep brain stimulation, neuromodulation, Parkinson’s disease, Alzheimer’s disease

## Abstract

Deep brain stimulation (DBS) is a surgical procedure that uses electrical neuromodulation to target specific regions of the brain, showing potential in the treatment of neurodegenerative disorders such as Parkinson’s disease (PD) and Alzheimer’s disease (AD). Despite similarities in disease pathology, DBS is currently only approved for use in PD patients, with limited literature on its effectiveness in AD. While DBS has shown promise in ameliorating brain circuits in PD, further research is needed to determine the optimal parameters for DBS and address any potential side effects. This review emphasizes the need for foundational and clinical research on DBS in different brain regions to treat AD and recommends the development of a classification system for adverse effects. Furthermore, this review suggests the use of either a low-frequency system (LFS) or high-frequency system (HFS) depending on the specific symptoms of the patient for both PD and AD.

## 1. Introduction

Alzheimer’s disease (AD) is a neurodegenerative disorder that affects millions of people worldwide, particularly the elderly. According to a WHO report in 2019, approximately 50 million people worldwide suffer from AD, with an incidence rate for dementia of 712 cases per 100,000 people globally in 2016 [1].

Most cases of AD are idiopathic, but studies have isolated certain risk factors that serve for the diagnosis of AD; these include mainly old age, genetic predisposition, and a healthy lifestyle (encapsulating dietary misbalance resulting in obesity and dyslipidemia, physical activity, marital status, and sleep) [2]. Moreover, other disease-related risk factors could include hypertension, cerebrovascular diseases, depression, and even microwave exposure [2,3]. The pathogenesis of AD is complex and involves several processes, including the hyperphosphorylation and oligomerization of tau protein, accumulation of amyloid beta (Aβ) plaques [4], and loss of cholinergic neurons and synapses [5]. These changes lead to progressive cognitive decline coupled with executive function depreciation as well as behavioral and psychiatric symptoms [4].

Parkinson’s disease (PD) is another neurodegenerative disease characterized by amyloid aggregation and deposition. It involves degeneration of dopaminergic neurons in the substantia nigra (SN) and intraneuronal formation of protein aggregates called Lewy bodies and Lewy neurites [6]. PD is a burdensome disease, with an annual incidence of 5–30 cases per 100,000 people globally [7]. Whilst most cases of PD are idiopathic, risk factors have been identified. Namely, genetic factors which increase susceptibility alongside rural living are most important, as well as pesticide usage and exposure to the chemical MPTP, which have been marked as significant risk factors [8,9]. More recently, other risk factors such as dairy consumption, cancer, usage of methamphetamine, traumatic brain injury, diabetes, cholesterol, alcohol, and hypertension have been linked to an increased risk of developing PD. Clinically, PD presents with progressive motor decline, including asymmetric resting tremor, cogwheel rigidity, and bradykinesia, as well as nonmotor symptoms such as anosmia, depression, and cognitive decline [10].

Despite their distinct clinical presentations, AD and PD share similar underlying pathophysiological mechanisms. Both are protein-misfolding disorders that result in the aggregation of misfolded amyloid proteins in neurons. In AD, tau (τ) and Aβ proteins are involved, while α-synuclein plays a major role in PD [11,12,13]. Additionally, both conditions are associated with dementia, and both PD and AD dementia have similar symptoms that may be relieved by deep brain stimulation (DBS) [12,14].

Current pharmacologic therapies for both disorders are primarily symptomatic and do not significantly impact the underlying disease processes [15,16]. These drugs can also have undesirable side effects in addition to being ineffective for large demographics of the patient population, especially younger patients and those who have worse clinical prognoses and disease progression. Therefore, there is a need for more effective disease-modifying therapies with a favorable side-effect profile. DBS is a potential treatment option that has shown promise in this regard.

This review will examine the development and current use of DBS in the treatment of PD with the aim of using the knowledge gained from studying PD to apply similar protocols in the treatment of AD patients. Currently, a large body of research points to similarities in the clinical pathologies of PD and AD, though studies have yet to draw a parallel between DBS use in PD and AD. As such, this review considers the potential of DBS as an effective treatment to both PD and AD patients, what has currently been found from the use of DBS as a licensed treatment for PD, and what translational qualities can be transferred to aid in the development of DBS for AD. Moreover, this review aims to highlight future developments that may improve the efficacy of the procedure and develop a series of recommendations for the use of DBS in the treatment of these two neurodegenerative diseases.

## 2. Search Strategy

The literature for this review was obtained from various sources including PubMed, Google Scholar, and Scopus, using search terms including “deep brain stimulation”, “Parkinson’s Disease”, “Alzheimer’s Disease”, and combinations of these terms. The literature was included in a narrative manner to highlight the development of DBS, its current application, and its future in the treatment of patients. Human trials were obtained from Google Scholar, Embase, and the United States National Library of Medicine’s clinical trials database. The information extracted included the region of implantation, laterality of the procedure, configuration parameters used for the DBS systems, duration of the study, number of patients involved, and current status of the study.

## 3. Deep Brain Stimulation

DBS is an invasive but established neurosurgical procedure that involves the implantation of one or more electrodes into a targeted brain area, an implantable pulse generator (IPG), and an extension connecting the electrode to the IPG [16]. The IPG contains the battery and circuitry, which generate the electrical signal that is delivered to the targeted brain structure. The DBS system allows for the delivery of electrical pulses to specific areas of the brain with minimal effects on nearby regions [17].

### 3.1. History of DBS

DBS is a relatively novel procedure, with most of the research conducted in the past 20 years. Prior to its development, surgical solutions were limited to ablation and the removal of affected brain regions, collectively known as craniotomy [18]. An example of such a procedure is thalamotomy, which entails the excision of the thalamus from the brain or sections of it [19]. While these procedures alleviated bradykinesia (slowness of movement) and dyskinesia (erratic excessive movement), they had major drawbacks including aphasia and dysphagia [20]. Even with the development of more precise surgical procedures such as stereo lesioning [21], the potential for adverse effects from the removal of brain tissue underscores the need for less invasive, better-tolerated therapies.

**Figure 1:**
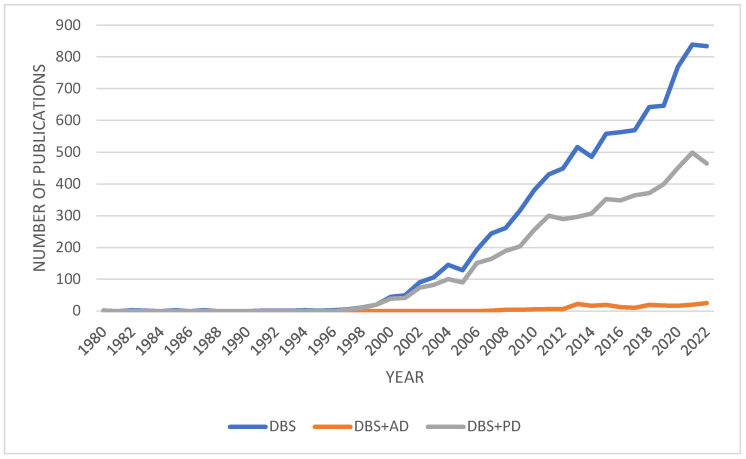
A graph of the number of research articles published on deep brain stimulation between 1980 and 2022. The queries performed were “DBS and deep brain stimulation”, “DBS and deep brain stimulation and AD and Alzheimer’s Disease”, and “DBS and deep brain stimulation and PD and Parkinson’s Disease”. Search parameters where limited to articles, books, and book chapters. Data extracted from Scopus: https://www.scopus.com (Accessed on 15 December 2022).

The use of DBS dates back to 1964, when Ohye et al. used a stereotactic procedure to stimulate the ventrolateral thalamic nucleus [22]. Since then, the volume of research on DBS has steadily increased (Figure 1). Initially, DBS was intended to treat PD and other movement disorders, and the FDA approved its use for PD and essential tremor in 1997 [23]. However, funding for DBS research declined following the development of levodopa in 1969, which may explain the lack of interest until the late 1990s [20]. The approval of thalamic DBS for the treatment of Parkinsonian symptoms in 1997 reignited interest in the field, and the potential applications of DBS in the treatment of other conditions such as AD, dystonia, epilepsy, and psychiatric disorders are currently being heavily researched [20]. As evident in Figure 1, while a significant amount of research has been conducted on the effectiveness of DBS treatment in PD, AD has received far less attention (Figure 1). Thus, further research on the potential use of DBS in AD is necessary.

### 3.2. Current Treatment Plans

DBS can be used to modulate almost all regions of the brain for numerous neurologic conditions. The treatment process begins with the implantation of wires that taper into electrodes in the brain using a precise stereo lesioning procedure [16,21]. Different brain regions can be stimulated to alleviate the effects of different conditions (Table 1). 

Once the electrodes are positioned into the desired area of the brain, a separate implantable pulse generator (IPG) device is implanted in the chest wall with leads running under the skin [16]. The IPG can be adjusted externally to deliver a range of frequencies, pulse widths, and amplitudes to the targeted brain region unilaterally and bilaterally [20]. Different frequencies have been used to target specific symptoms, with high frequency stimulation (HFS) defined as above 100 Hz and low frequency stimulation (LFS) as 60–80 Hz [57,58,59]. However, this is quite subjective to each disease, as some papers cite that the range of 10–70 Hz is generally avoided due to the risk of eliciting seizures for epilepsy studies [59]. Some studies have found that frequencies around 60 Hz have restorative effects on PD symptoms, such as reducing aspiration and improving gait [25,60]. In particular, it is noted by di Blaise et al. that HFS systems decrease the levodopa-responsive PD symptoms, whereas LFS systems decrease more axial symptoms of the disease [58]. Indeed, Ramdhani et al. found that PD patients who experienced worsening of their symptoms after receiving HFS DBS had improvements in freezing of gait and dyskinesia when they were switched to a 60 Hz LFS system [60]. Contrastingly, Wyckhuys et al. discovered that HFS systems of 130 Hz are more effective at increasing the threshold and latency of after-discharges in kindled rats, a common animal model of epilepsy [59]. These studies suggest that the use of HFS and LFS systems is dependent on the type of disease that is to be treated, as well as the specific symptoms to be alleviated. The next section details the exact surgical methods used to implant the DBS system in patients.

### 3.3. Patient Screening

Given the wide range of neurological disorders that can be treated by DBS, it is important to carefully select candidates for the treatment. One important factor to consider is the length of time since the patient’s diagnosis. There is conflicting evidence whether DBS is more effective for patients who were diagnosed within the past 5 years or for those who were diagnosed earlier. For PD, some studies suggest that earlier treatment may be more beneficial [61], while others suggest that it is important to monitor patients over a longer period of time to identify atypical symptoms and assess treatment suitability [62]. Moreover, surgically invasive procedures are usually left as a last-resort treatment and thus are usually used on older patients who have been diagnosed for longer. However, this approach benefits from the ability to rule out other “Parkinson-plus” diseases which may not be clinically treatable through DBS, such as comorbidities of PD and other diseases with similar symptoms [61].

Patients being considered for device-assisted therapies must undergo evaluation by a specialized center’s multi-disciplinary team, which typically includes a functional neurosurgeon, an anesthesiologist, a movement disorder neurologist, and a neuropsychologist, as well as representatives from departments such as radiology, psychiatry, physical therapy, nursing, and social work.

Another factor to consider when selecting candidates for DBS is the patient’s responsiveness to levodopa, a common medication used to treat PD. Many studies have found that patients who are responsive to levodopa are more likely to also respond to DBS [62,63], while some others have found conflicting evidence [64,65]. The site of stimulation may affect the relationship between levodopa responsiveness and DBS efficacy, but the reason for this is not well understood. Lin et al. found a positive correlation between levodopa responsiveness and GPi-DBS efficacy (R^2^ = 0.283, *p* = 0.016), though STN DBS was more efficacious than GPi-DBS for levodopa-resistant tremor control [66]. There is no current indication of how medication for AD may interact with DBS treatment, but co-therapy to mitigate the side effects is certainly something future clinical trials could investigate. In the future, online tools may be developed to help improve the selection process for DBS candidates and provide more uniformity in the selection process. One study found that the use of an online tool called Stimulus to screen 3128 patients led to a significantly higher acceptance rate than conventional multi-disciplinary screening [67]. 

### 3.4. Procedure and Mechanism of Action

The DBS hardware includes multiconductor intracranial quadripolar electrodes, a programmable single- or dual-channel internal pulse generator with battery unit, and an extension cable connecting the DBS electrodes to the pulse generator [20]. 

**Figure 2:**
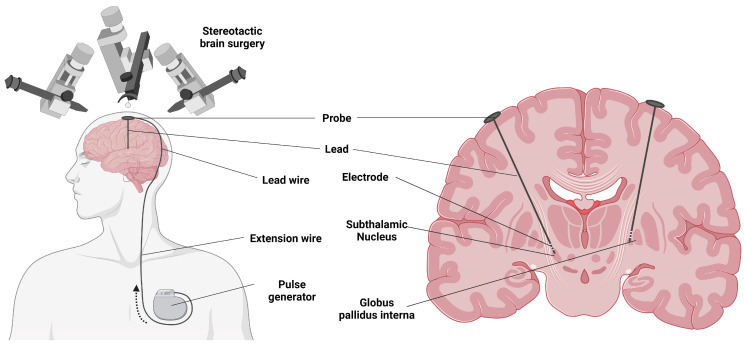
Electrode implantation for deep brain stimulation in Parkinson’s disease. Created using https://BioRender.com (Accessed on 19 December 2022).

The procedure involves stereotactic placement of electrodes in the target area, either unilaterally or bilaterally (Figure 2). This is the first stage of the procedure and is often performed while the patient is awake. In the second stage, the electrode and extension cable are tunneled under the skin to the infraclavicular area, where they are connected to the battery-powered pulse generator [44].

When activated, the pulse generator delivers electric stimulation to the targeted area. The exact mechanism of action is not fully understood, but it is hypothesized that high-frequency DBS in PD suppresses GPi neuronal activity and efferent fiber pathways. This disrupts the flow of abnormal information through the cortico-basal ganglia circuits and downstream pathways, improving symptoms [68,69].

Overall, DBS as a procedure has come a long way since its initial development in the 1960s. Moreover, it is increasingly being trialed as an efficacious invasive procedure to alleviate the symptoms of many diseases. Additionally, more data are being generated on patient criteria which allows them to undergo ameliorative DBS treatment as well as the potential mechanisms by which DBS may affect different neurodegenerative diseases. Currently, the DBS only has regulatory approval in the treatment of PD, which will be explored in the next section.

## 4. DBS in Parkinson’s Disease

PD is a complex and progressive neurodegenerative disease that is primarily characterized by motor symptoms. These motor symptoms may include a slowly progressive asymmetric resting tremor, stiffness in the muscles (cogwheel rigidity), and difficulty with movement and coordination (bradykinesia). Other common nonmotor symptoms associated with PD include loss of sense of smell (anosmia), constipation, depression, and sleep disorders. Autonomic dysfunction, pain, and cognitive decline may occur in the later stages of disease [10]. This section explores our current understanding of PD pathophysiology and how DBS has been found to affect its mechanisms at a pathological and symptomatic level. Moreover, the parameters for use of DBS on patients and the effect of age are discussed, along with currently identified adverse effects of treatment.

### 4.1. Pathology of PD

In terms of neuropathology, PD involves the degeneration of dopaminergic neurons in the substantia nigra (SN) and the presence of intraneuronal protein aggregates called Lewy bodies and Lewy neurites [6]. Recent studies have suggested that loss of dopaminergic terminals in the striatum, rather than the SN, may be more responsible for the motor symptoms observed in PD [70]. Lewy pathology, which is the microscopic evidence of the misfolding of the α-synuclein protein [71], is a characteristic finding in most cases of PD (some rare genetic forms of PD involve the loss of striatal dopaminergic neurons without these protein aggregates [72]). α-synuclein protein is commonly found in synapses, where it plays a role in the function of synaptic vesicles; it is also found in non-neuronal cells such as hepatocytes, myocytes, lymphocytes, and erythrocytes, although its functions in these cells are not yet fully understood [73].

Braak and colleagues proposed a six-step pathological pathway for Lewy pathology in PD; the first stage is limited to the dorsal motor nucleus of the vagal nerve, while the proteins progressively spread to the rest of the brain in the subsequent stages [74]. These conclusions were based on post-mortem observations and do not have contemporary evidence to support them; indeed, recent observations suggest that clinically diagnosed PD patients may have a different pathology compared to what the Braak stages describe [75]. Nonetheless, Braak et al.’s model has gained popularity in recent years, and the earliest stages of Lewy pathology (before the aggregates reach the SN) are thought to be linked to the signs and symptoms of premotor PD [75].

Different α-synuclein assemblies can be secreted by neurons, and this process is upregulated if the lysosomal–autophagy system is inhibited. These assemblies can then be taken up by nearby neurons, where they can seed monomeric α-synuclein into Lewy-like aggregates. This prion-like property of α-synuclein assemblies may explain how Lewy pathology propagates between brain regions [76]. However, the cause of this aggregation is unknown. One theory is that environmental substances such as pesticides and pollutants, as well as pathogens, can enter α-synuclein-containing cells in the olfactory system and gastrointestinal tract. In a healthy individual, these aggregates would be mitigated by cellular proteostatic mechanisms and not lead to the spread of Lewy aggregates; however, in the presence of factors such as aging, genetic predisposition, and peripheral inflammation, it is proposed that α-synuclein may bypass the cell’s normal clearance mechanism and cause Lewy aggregates to form in the brain [77].

### 4.2. Current Treatments for PD

While there is currently no truly disease-modifying treatment for PD, there are several drug therapies available for symptom management [78]. These include dopamine medication, the most common of which is levodopa. These dopamine agonists can be taken orally or through injection and are a preferred treatment for PD patients, especially in the later stages of the disease. However, levodopa loses effectiveness over time, requiring increasing dosage, and can cause side effects [78]. There are also monoamine oxidase inhibitors (MAOIs) and antagonists that can be taken orally and function to prevent the breakdown of levodopa and dopamine [79]. These drugs do not need to have their doses increased over time and have milder side effects compared to levodopa. However, these are not as effective in later stage PD and usually need to be supplemented with another group of drugs that have their own associated symptoms [78].

Another set of oral medications, anticholinergics, reduces acetylcholine activity at choline receptors and can be used as a monotherapy in the early stages, but there is reduced tolerance in elder patients and limited pharmacokinetic information available [80]. In addition to these medications, some surgical treatments, such as ablative surgery, are also used, although their side effects and symptoms may not be reversible [78].

### 4.3. Use of DBS in the Treatment of PD

The surgical treatments of PD have evolved over time. The early 1940s saw surgical procedures focused on lesioning the thalamus and GPi. These treatments, known as pallidotomy, became common before the use of levodopa became widespread. Later, surgical procedures saw a resurgence due to the side effects of levodopa. In the 1990s, DBS emerged as a replacement for lesioning treatments due to the adverse effects associated with bilateral lesions and the irreversible side effects of poorly placed lesions [81].

**Figure 3:**
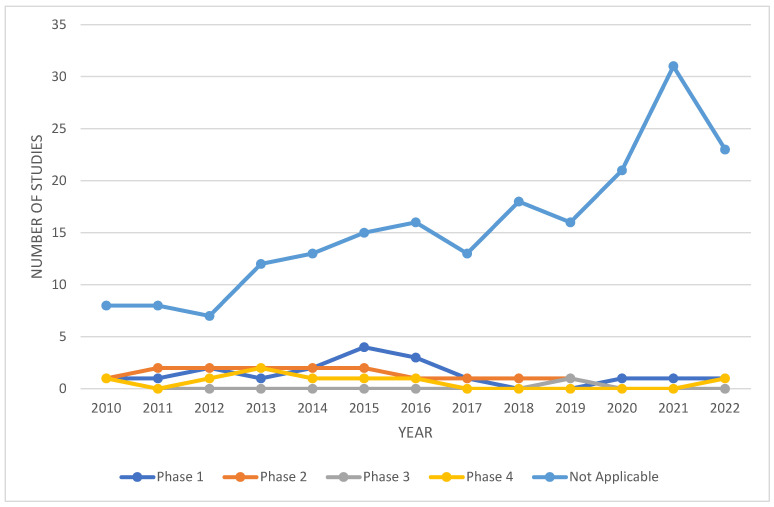
A summary of clinical trials that have been completed or are currently active on DBS and PD. **Phase I trials** focus on the safety of a procedure and involve a small number of healthy volunteers. They are designed to determine the most serious and common adverse side effects. **Phase II trials** are the first time that a procedure is tested on the relevant patient population. Adverse side effects continue to be evaluated. **Phase III trials** involve a larger population of patients and are used to evaluate the correct doses and criteria for successful use of a procedure for the relevant affected population. **Phase IV** trials are conducted after a treatment has been approved by the FDA and are used to continue to evaluate the efficacy, best dose, and safety of the approved treatment. **Not applicable**: these trials do not fit into the FDA-defined phases and may include trials of devices or behavioral interventions. All data were collected from https://www.clinicaltrials.gov/ (Accessed on 20 December 2022).

There have been many clinical trials that have studied the use of DBS in the treatment of PD (Figure 3). Some of these trials have compared DBS combined with medical therapy to medical therapy alone [82], while others have focused on the differences in effects seen when different brain regions are stimulated [83]. Moreover, several clinical trials have looked at how DBS treatment for PD may affect patients of different ages [84]. These studies combined seem to suggest that DBS can be an effective therapy for PD [85]. However, it is worth noting that many of these trials are not double-blind and have relied on open-label data from individual institutions [85]. Further clinical trials, with more objective rating scales and controls, could provide stronger evidence for the effectiveness of DBS in PD treatment.

DBS can be used to stimulate different brain regions in PD patients, and each can have different effects on the symptoms of the disease. Stimulation of the GPi may reduce motor symptoms [86] and also alleviate painful dystonia and sensory symptoms, but it does not usually allow for a reduction in medication usage [87]. Stimulation of the STN can reduce the need for dopaminergic medication [88] and also improve gait, tremor, and bradykinesia [89,90]. However, stimulation of the STN may not address all of the major motor symptoms of PD.

### 4.4. Mechanisms of Action of DBS on PD

The exact mechanism by which DBS works in the treatment of PD is not fully understood and is a subject of ongoing research and debate [91]. Ongoing studies are attempting to understand the communication between brain structures at various levels (subcellular, neuronal, and fiber-pathway levels), how DBS modulates dysfunctional pathways in the brains of PD patients, and the plastic changes that occur in the brain due to DBS [92]. While there is still much to learn about the mechanisms of DBS in PD, some theories have been proposed [92]. One theory suggests that DBS works by inhibiting the activity of target neurons through a variety of means such as blocking the transmission of nerve impulses, disrupting the release of neurotransmitters such as glutamate, or increasing the release of inhibitory neurotransmitters such as GABA and adenosine [92,93,94,95]. Another theory proposes that DBS activates target neurons by increasing the levels of certain neurotransmitters such as glutamate [96,97]. A third theory suggests that DBS may both inhibit and activate target neurons by affecting the communication between the cell body and axons of neurons [70,71]. Additionally, DBS may disrupt abnormal oscillatory patterns by replacing irregularly firing cells with regularly firing cells, producing “jamming” signals that can lead to the release of neurotrophins and the generation of new neurons [98]. The varied and sometimes conflicting results of studies on the mechanism of DBS may be due to the many experimental variables that can affect research outcomes, such as the different approaches, types of stimulation, and time intervals at which the effects are observed.

### 4.5. Criteria for Successful Treatment of DBS in PD

The success of DBS for treating PD is highly dependent on careful selection of patients. Over 30% of DBS failed attempts are attributed to an inadequate selection process [99]. According to a 2007 survey, young patients with (1) good levodopa response, (2) no or few axial non-levodopa motor responsive symptoms, (3) few or mild cognitive impairments, and (4) absent (or well-controlled) psychiatric disease are best suited for DBS treatment for PD [100]. Admittedly, strict adherence to these criteria may exclude many patients who could still benefit from the surgery. The most reliable predictor of success with DBS is patients’ response to levodopa [101]. A 30% improvement in the Unified Parkinson’s Disease Rating Scale III (UPSDIII) score is commonly used as a marker of levodopa responsiveness, with severe tremor resistance to levodopa therapy being an exception [102]. Patients with atypical forms of parkinsonism and dementia tend to have less favorable outcomes with DBS [102,103]. Additionally, DBS surgery is delayed for patients with psychiatric symptoms, until their symptoms are properly managed [81]. Furthermore, the prior experience of the surgical team is also a key factor in determining the outcome of DBS surgeries. It is recommended that DBS programming is best performed by a highly trained clinician with expertise in both DBS technology and PD-related issues as well as pharmacological management [103].

### 4.6. Parameters for DBS use in PD

The optimal parameters for DBS in treating PD are not well understood and can be challenging to determine. Some studies suggest that a frequency around 60 Hz is best for use in PD [25,60], but there is still debate among experts in the field. Currently, the widely accepted range for treating parkinsonian symptoms is between 130 and 180 Hz [104,105], though emerging research has shown that long-term HFS treatment may not treat axial symptoms of the disease or even be deteriorative [106]. A consensus of experts has agreed that the relevant parameters of pulse width, frequency, voltage, and electrode configuration need to be optimized within 3 to 6 months during four to five programming sessions to ensure maximum benefit to patients [81]. However, finding the optimal parameters is considered as much of an “art” as it is a science [92]. There are several ways to improve the process of creating optimal parameters [92], including careful imaging to identify the exact electrode contacts in the brain, modeling the spread of electrical fields [98], identifying physiological changes associated with stimulation that show promise for long-term benefit, and automating the optimization process to reduce the risk of error and the time required [107].

### 4.7. Effect of Age on the Effectiveness of DBS on PD

The appropriateness of DBS as a treatment option for Parkinson’s disease in elderly patients has been a topic of debate [81]. Historically, DBS has been considered a viable treatment option for patients under the age of 70, while those older than 70 have been excluded. However, Mathkour et al. evaluated the short- and long-term outcomes of DBS in patients with PD who were older than 70 years old and underwent DBS [108]. The study found a significant decrease in UPDRS III score (preoperative 31.8 to postoperative 15.6; *p* < 0.0001) as well as a significant reduction in medication doses per day (preoperative 11.54 to postoperative 7.97; *p* = 0.0112) [108].

A separate study compared the long-term outcomes of DBS in Parkinson’s disease patients that were young and old [109]. Both groups experienced a significant decrease in levodopa-equivalent dose daily (LEDD), with the elderly group seeing a more significant decrease [109]. Moreover, both groups had a reduction in UPDRS III score, which in both groups indicated significant improvements in motor function. Furthermore, elderly patients experienced a greater reduction in daily doses compared to the younger group. These results indicate that DBS can be beneficial for both younger and older patients [109]. A higher incidence of negative side effects or follow-up issues with older patients may be attributed to age-related comorbidities such as cognitive decline [102], a higher incidence of levodopa-resistant symptoms [84], and a higher overall risk of surgical complications [7].

### 4.8. Adverse Effects of DBS in PD

Currently, there exists no unified framework in which to describe the adverse effects of DBS, though some studies have tried to create such a system and put it into practice [110]. However, adverse effects can definitely be seen with the use of both STN and GPi DBS. Broadly, these issues can be categorized into procedure-related, hardware-related, and stimulation/disease-progression-related issues [110].

Procedurally, surgical implantation has its risks given the invasiveness of the procedure. Indeed, complications of the procedure may include intracranial hemorrhage (0–10%), stroke (0–2%), infection (0–15%), lead erosion without infection (1–2.5%), lead fracture (0–15%), lead migration (0–19%), and death (0–4.4%), according to Bronstein et al. [81]. Overall, STN-DBS has demonstrated a higher rate of surgical complications in the studies assessed by Videnovic et al., though this may be attributed to the larger sample size of patients who underwent STN-DBS treatment [110].

In terms of disease progression, patients with STN-DBS required less dopaminergic agents than those who received GPi-DBS. However, patients undergoing GPi-DBS showed an improvement in levels of depression post-operatively and decreased loss of visuomotor processing speed than STN-DBS patients in the long term [81,111]. Moreover, patients also reported a high incidence rate of weight gain (37.5%), speech disturbance (12.8%), eyelid opening apraxia (11.3%), and cognitive decline (5.8%) in GPi-DBS, though STN-DBS patients reported an uncharacteristically high gait ignition failure rate (17.6%) [110].

Long-term effects of DBS on PD include improvement in motor fluctuations and tremors for up to five years [112,113,114], but eventually patients may develop symptoms that are levodopa-resistant including freezing of gait, postural instability, and cognitive decline [81]. As such, it can be said that both forms of treatment have their individual advantages and drawbacks, though more research is required into GPi-DBS and its adverse effects to have a fair comparison between the two types of therapies.

In summary, DBS has shown versatility in the treatment of patients with PD. The GPi and STN regions have been identified as positive targets for DBS therapy. Moreover, there is a growing body of research which highlights the effect of DBS on the pathology of PD as well as marked improvements in the symptoms of patients who have undergone DBS treatment. While broadly applicable standards for settings to use in treatment and age eligibility criteria still remain elusive, the identification of possible side effects and their mitigation has allowed DBS to be established as a versatile technology in the treatment of PD. As such, this knowledge base provides the foundational basis to explore the use of DBS in AD treatment, which will be discussed in the next section.

## 5. DBS in Alzheimer’s Disease

AD is a neurodegenerative disorder that affects memory and cognitive functions. It is the most prevalent type of dementia, accounting for the majority of dementia cases worldwide [115]. AD is characterized by a gradual decline in memory and cognitive abilities. Imaging studies, such as magnetic resonance imaging (MRI), can show shrinkage of the hippocampus, an area important for memory. Additionally, biomarkers such as decreased levels of the protein amyloid β42 in the cerebrospinal fluid and the presence of phosphorylated τ proteins detected by positron emission tomography (PET) can also indicate the presence of AD [116]. This section explores our current understanding of AD pathology, conventional methods of treatment, and what DBS has to offer. Moreover, selection criteria for patients and potential adverse effects found in the present literature are also reviewed.

### 5.1. Pathology of Alzheimer’s Disease

AD and PD are similar in that both are caused by protein misfolding. In AD, the aggregation of extracellular Aβ and intracellular hyperphosphorylation of τ protein leads to the formation of plaques and neurofibrillary tangles (NFTs) [13]. This results in the loss of cholinergic neurons and synapses [115].

The cause of Aβ aggregation is not fully understood, but genetic factors such as missense mutations in the genes encoding amyloid precursor protein (APP), presenilin 1 (PSEN 1), presenilin 2 (PSEN 2), and apolipoprotein E (APOE) are known to increase the risk of AD [13,115]. In particular, early onset AD is often inherited and caused by mutations in the APP, PSEN 1, or PSEN 2 genes [13]. While there is ongoing debate as to what these genes code for, it is well established that these mutations can lead to γ-secretase forming more Aβ fibrils [117]. Late-onset AD is associated with mutations in the APOE gene, which increases the risk of vascular dementia, Lewy body dementia, and others [115].

Regardless of the cause, the overproduction of Aβ leads to its aggregation as plaques extracellularly, though the exact mechanism of its exocytosis remains unclear [118]. This leads to the hyperphosphorylation of τ protein by the dysregulation of kinases such as CDK-5 and GSK-3β by Aβ fibrils [119]. This in turn leads to conformational changes in τ, resulting in the formation of NFTs inside the neuron [119]. Aβ also triggers immune responses in the microglial cells through toll-like receptors, leading to inflammation, receptor-mediated phagocytosis, and cell clearance [120]. These mechanisms ultimately result in decreased brain weight and neuronal loss, particularly in the white matter and hippocampus [115]. DBS is being evaluated as a potential treatment to address these mechanisms.

### 5.2. Current Therapeutics in AD

The primary physiological feature finding of AD is the presence of NFTs and Aβ fibril plaques. As a result, most currently FDA-approved drugs aim to prevent their formation, thereby reducing symptoms. Currently, the FDA has approved three acetylcholinesterase enzyme (AChE) inhibitors: donepezil, galantamine, and rivastigmine, as well as one N-methyl-D-aspartate (NMDA) receptor antagonist: memantine [121,122].

AChE inhibitors function by addressing cognitive dysfunction in AD caused by the loss of cholinergic nerves in the brain [123]. They perform this by inhibiting the enzyme acetylcholinesterase that breaks down acetylcholine, leading to increased levels of the neurotransmitter in cholinergic neurons [122]. This approach has been shown to be effective in clinical trials but may only halt or slow the rate of cognitive decline and not address underlying neuronal loss and brain atrophy [124,125]. NMDA receptor antagonists, on the other hand, prevent excitotoxicity and cell death by inhibiting the rapid influx of Ca^2+^ ions into neurons [121]. They can also mediate neurotoxicity induced by the presence of glutamate, thereby preventing neuronal death [122]. However, this treatment is typically used for mild to moderate cases of AD [121].

Recently, the FDA has also partially approved a drug called Aducanumab developed by Biogen, which has been somewhat controversial [126,127]. This drug involves injecting monoclonal antibodies that bind to Aβ and its aggregates, allowing for their removal by the body [122]. This is the first disease-modifying therapy approved for AD patients [127]. However, some studies have pointed out that that this drug focuses heavily on the Aβ aggregate pathology without taking into consideration τ protein aggregation in NFTs, and the effects on cognitive decline in phase III trials were limited [122,126]. Unfortunately, anti-τ therapies are mostly still in the early stages of clinical research, and it is uncertain how they will fare in further testing [122]. As such, an alternative approach to address AD has been found in DBS treatments.

### 5.3. Use of DBS Treatment in AD

DBS treatment for AD is not yet approved by the FDA or other regulatory bodies. However, several clinical studies are being conducted to better understand the effects of DBS on humans. These studies primarily focus on stimulating specific areas of the brain such as the fornix, hippocampus, and nucleus basalis of Meynert (NBM). The results of these studies are summarized in Table 2:

Overall, the clinical trials presently completed paint a positive outlook for the use of DBS in the treatment of AD. Specifically, phase I and II trials conducted on AD patients using bilateral fornix DBS have been shown to have positive outcomes on symptoms of AD [34,37]. Moreover, present clinical trials have also shown good tolerance and a lack of negative outcomes from the bilateral stimulation of the NBM region. Most importantly, DBS treatment seems to have halted or even reversed cognitive decline among patients undergoing treatment, highlighting a potentially bright future for the use of NBM-DBS in the treatment AD patients [38].

Additionally, only one trial has investigated the use of DBS to stimulate the hypothalamus, which has been found to be heavily affected by AD pathology [134]. There is also a lack of research using in vivo techniques in rat models to study the effectiveness of DBS in treating AD symptoms, although some studies have shown promising results for fornix DBS in transgenic mice models [138,139]. Research on the neuroprotective effects of DBS on the NBM has also been conducted in rat models with positive results [140,141]. Therefore, further research using in vivo techniques to identify suitable regions of the brain for DBS and progression of existing clinical trials for fornix DBS would be beneficial in obtaining approval for DBS as a treatment for AD. Additionally, understanding the underlying mechanism of how DBS affects AD pathology would help in identifying areas of the brain to target, which will be discussed in the next section.

Many of the completed studies above are relatively outdated and have small sample sizes, with the other clinical trials currently only in the recruiting phase. Moreover, many studies have yet to disclose certain vital information regarding the procedures conducted such as laterality and the specific settings used for stimulation therapy. As such, further transparency in the clinical studies in progress and further exploration of other potential sites for DBS stimulation would go a long way to advance the present paucity in the literature.

### 5.4. Mechanisms of Action of DBS in AD

DBS can affect the underlying pathology of AD in various ways, although the majority of these mechanisms are still being researched. Currently, it is known that DBS increases neuronal activity in the Papez circuit of the brain by activating neurons in the hippocampus, parahippocampal gyrus, and default mode network (precuneus, parietal, and temporal lobe) [142]. The most common target for DBS in AD is the fornix within the hippocampus, which has been shown to increase glucose metabolism and utilization in the cortico–thalamic and cortico–hippocampal networks [143]. This is associated with better clinical outcomes and also mitigates neuronal loss and synapse reduction [144], leading to greater hippocampal volume [142].

Stimulation of the NBM appears to have similar effects on fornix DBS. Cholinergic neuron degeneration in the medial forebrain is a characteristic sign of AD. Multiple phase I clinical trials have shown that stimulation of the NBM can result in a slowed increase their Alzheimer’s Disease Assessment scale (ADAS) scores and stabilization of Mini-Mental State Examination (MMSE) or Clinical Dementia Rating (CDR), increased cortical glucose uptake, and decreased motor disability [38,145]. Additionally, it is hypothesized this may be the result of increased acetylcholine levels in the cortex, leading to improved cognitive functions [38].

A third potential treatment of DBS in AD is the stimulation of the ventral capsule/ventral striatum (VC/VS), but data on this are limited. Jordan et al. report on a phase 1 clinical trial that showed increased prefrontal glucose metabolism and decreased clinical decline as compared to patients without VC/VS DBS [146]. Unfortunately, no further trials have been conducted in this area. More research is needed to understand the underlying mechanism by which AD pathology is affected by DBS and how it can be modulated through treatments.

### 5.5. Criteria for Successful Applications of DBS in AD

The regulation of selecting viable candidates for DBS treatment of Alzheimer’s disease is not well established. Studies have used a variety of selection criteria, such as ADAS scores, MMSE, and CDR.

The ADAS is a cognitive assessment used to quantify the extent of disease progression among AD patients [147]. Moreover, the practicality of ADAS in clinical assessment has also been noted in previous studies [148]. The MMSE is a similar but more wide-ranging test meant to assess the severity of cognitive impairment and serves as a quantifiable metric to judge cognitive decline [149]. Clinically, the ADAS scale has been used in conjunction with other tests such as the MMSE and shows significant correlation between the two tests [150]. The CDR scale is also comparable with the MMSE and ADAS, though it is a standardized scale which tests the extent of progression of dementia among afflicted patients [151].

Depending on the study, different selection criteria have been used to assess the suitability of candidates. The Advance study group led by Lozano et al. is the most recent group of researchers to conduct clinical trials into the effectiveness of fornix DBS on AD [34]. Their study selected patients with mild AD who had CDR scores of 0.5–1 and ADAS scores of 12–24 [34]. However, other studies used the MMSE to assess the extent of AD progression. The study led by Fontaine Denys et al. required the patient to have an MMSE score between 20 and 24, which is indicative of mild cognitive decline [134], yet other studies used none of the above metrics and instead chose to use the criteria of the National Institute of Neurological and Communicative Disorders and Stroke–Alzheimer’s Disease and Related Disorders Association (NINCDS-ADRDA) [130]. Consequently, it is difficult to draw any definitive conclusions regarding the efficacy of DBS interventions in AD patients.

It is important to note that the metrics used to evaluate the effectiveness of AD diagnosis are outdated. Newer technology and standards may be more accurate [152]. A review of the current diagnostic tools and clearer criteria for selecting AD patients for DBS clinical trials is needed. Additionally, the age of the patient should also be considered.

### 5.6. The Effect of Age on the Effectiveness of DBS in AD

As with other areas of DBS research into AD, age and its correlation with DBS efficacy has not yet been well documented. Many of the studies listed in Table 2 focused on a narrow age range of patients. For instance, Fontaine et al.’s current study included patients between 50 and 65 years old, while Laxton et al.’s study required patients to be between 40 and 80 years old, but only had six patients between the ages of 51 and 68 [37,134].

The study conducted by Lozano et al. during the Advance phase II trials is the only research found to investigate the varying effects of DBS on different age groups. Their study, which involved fornix DBS, revealed that patients under 65 years of age experienced a deterioration in MMSE and CDR scores with the use of this technique [34].

Subsequent review by Lozano et al. and analysis by Aldehri et al. attributes the smaller age range used to a difference in the pathophysiology of AD between younger versus older populations, leading to a difference in clinical efficacy [34,153]. To this end, Lozano et al. suggest that the lack of ameliorative effects from DBS may be due to a more severe disease progression among younger patients, even though clinical symptoms may be similar to older patients. Indeed, a broader review by Schneider et al. showed that younger patients with AD are at greater risk of cognitive decline than older participants over a period of 12–24 months [154]. Conversely, this may also be due to different genetic predispositions which skew the results, given that only a small sample size of patients was used [34].

Consequently, the need for younger patients to receive DBS treatment in different regions of the brain is imperative. Moreover, further research into the reason behind the lack of DBS efficacy in younger patients should also be explored. In line with this finding, more recent research conducted by Lozano et al. has adopted larger age ranges of 45–85 years old [34].

### 5.7. Adverse Effects of DBS in AD

The present dearth in clinical trials with the use of DBS in AD makes it very difficult to discern possible adverse effects of the treatment. Certainly, none of the completed trials report any adverse effects related to hardware malfunction or manifestation of psychiatric complications.

However, the incidence of side effects post-operatively and changes to disease progression have been reported in the clinical trials. Laxton et al. reported that some patients experienced stimulation-induced autobiographical memory recall with a general warm, flushing feeling [37]. This may be due to stimulation of the fornix, which is not the primary target of treatment. Moreover, patients also increased heart rate and systolic blood pressure during stimulation [37]. In terms of clinical outcome, most patients were reported to have a decreased ADAS score in the short term, which were predicted to increase to pre-operative levels after 12 months post-operatively. Within this cohort, one patient did show improvement in their MMSE score but a deterioration in ADAS score [37]. Other studies such as Mao et al. and Kuhn et al. corroborate these findings with having patients in their cohorts that showed minimal amelioration of their ADAS and MMSE scores or even worsening of it post-operatively [33,38]. Mao et al. reported one of their patients also having worsening clinical presentation with a degeneration of his basic activities of daily living [33]. Moreover, Kuhn et al. also reported stimulation-induced inner restlessness in one of the patients of their study [38].

The outlook for using DBS to treat AD appears promising. Despite ongoing research into the precise mechanism of AD pathology, DBS may be a highly viable alternative to conventional pharmaceuticals, which often come with unappealing side effects. However, it is unfortunate that there is still some uncertainty or limitation surrounding its efficacy and safety. There is definitely a shortage of research into the use of DBS for AD in both animal models and humans. Only the fornix and NBM have been identified as potential targets in ongoing or completed human trials. Therefore, further efforts are needed to understand the specific criteria for selecting AD patients who could benefit from DBS treatment, the impact of DBS on AD pathology, and the potential adverse effects associated with DBS. These are crucial steps needed to fully integrate DBS as a frontline treatment for AD.

## 6. Discussion

Current research suggests a strong connection between AD and PD, as well as the effectiveness of DBS in treating the symptoms of both conditions. Both diseases result in neuronal loss through different mechanisms [6,115]. Moreover, many PD patients also develop AD within 20 years of diagnosis, highlighting the importance of treating co-occurring conditions simultaneously, such as AD in PD [155].

As noted in previous sections, the current application of DBS in PD has had positive responses. While we are still exploring the exact pathology of the disease, it is known that Lewy body aggregates are the primary cause [72]. Moreover, conventional therapies used in PD have been shown to have many adverse effects and a lack of efficacy at advanced stages of the disease [78]. Consequently, the use of DBS in treatment has allowed for better coverage and versality in treatment. Additionally, while current pathologies and mechanisms of action of DBS are listed below in Table 3, there is much to be learned in the action of multiple comorbidities on the human body.

It is important to note that the proposed mechanisms of action outlined above in Table 3 pertain to known human trials, while animal studies have revealed additional mechanisms. For example, DBS treatment has been found to decrease amyloidosis, inflammation, neuronal loss, pathological τ-protein formation, and cholinergic nerve degeneration in animal models [138,157,158]. Other mechanisms such as synaptic plasticity, τ clearance, increased neurotropic factors, and increased tyrosine hydroxylase (TH) in substantia nigra pars compacta (SNpc) neurons have also been observed in animal models but have yet to be extensively tested in humans [142,159].

Studies on both AD and PD use a combination of quantitative and qualitative tests to determine eligibility for DBS treatment. A multi-disciplinary team is also involved in the selection process for both diseases. Age is also an important factor to consider, with younger AD patients being more at risk of cognitive decline [154] and older PD patients being more likely to experience adverse effects associated with age-related comorbidities [7].

**Figure 4:**
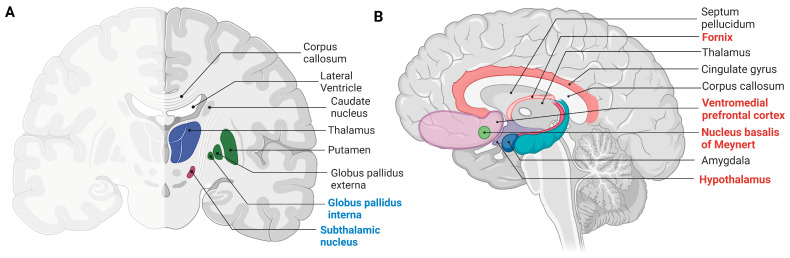
Anatomical representation of areas of the brain targeted by DBS treatment. (**A**) Frontal coronal cross-section of the brain depicting regions of the brain targeted for treatment of PD (highlighted in blue). (**B**) Frontal sagittal cross-section of the brain depicting regions targeted for treatment of AD (highlighted in red). Figure made and compiled using https://biorender.com/ (Accessed on 20 December 2022).

DBS is used to treat PD and AD, but the specific targets vary between the two diseases (Figure 4A,B). Studies have shown that DBS of the STN and GPi has similar effects on motor function in PD, but one study found that STN DBS may lead to faster cognitive decline [24,69,86,160]. Moreover, it seems that fornix-DBS is effective at combating hippocampal and forniceal volume degeneration. Further research is needed to determine which target leads to improved clinical outcomes.

There is limited research on DBS for AD, but the most promising results have been found with fornix and NBM stimulation [153,161]. It is suggested that while DBS of the fornix is more effective at slowing the degeneration of the hippocampus, DBS of the NBM targets the loss of cholinergic neurons [162].

Both diseases require high-frequency DBS, but more research is needed on the effects of DBS at different frequencies, amplitudes, and pulse widths, as well as potential side effects. The currently used frequencies are more based on trial and error rather than observable data sets and would benefit from the use of more automated systems [107].

Overall, DBS is a promising treatment for PD and AD by targeting the underlying pathologies of the diseases. However, there is still room for advancement to minimize adverse effects and maximize the benefit of DBS. Suggestions for improvements include developing new DBS leads that improve performance, minimizing complications from errant electrode placement, and reducing programming time with AI-guided parameter selection [81]. The pulse generators could be made smaller, with longer battery life, allow for situation-based stimulation patterns, and be shielded from electromagnetic interference. It is recommended to systematically test the effects of stimulation for all electrodes during the initial programming session, gradually reduce anti-PD medications, and use lower-frequency stimulation and alternative electrode configurations if problems are not adequately treated.

Advancements in the field also include the potential use of functional magnetic resonance imaging (fMRI) and machine learning (ML) in combination with DBS. A review of 83 studies found the fMRI and DBS combination to be a powerful tool for observing and manipulating neuronal circuits simultaneously [163]. ML has the potential to improve DBS outcomes [164], with a systematic review of 73 studies finding that ML can help process electrical and imaging data, though many challenges remain [165]. A recent trial showed the potential of ML and fMRI to complement each other, with fMRI serving as a biomarker of clinical response in DBS [166].

Future use of DBS may also have ethical implications that may impact its widespread application for PD and AD. These include the informed consent of patients with neuropsychiatric symptoms, informed consent given by guardians or caregivers, and guidelines to ensure safe and effective use across all centers and professionals [92,167]. Addressing issues of expertise and accessibility will ensure that DBS is accessible to all, regardless of their location or socioeconomic status [168]. Additionally, as DBS technology advances, policymakers must consider the potential impact on a person’s personality [167] and whether it is ethical to use DBS on “healthy” individuals for desired effects [169].

Certainly, the relative success of using DBS in treating PD can offer guidance for utilizing this treatment in other neurodegenerative diseases, particularly in AD. Both diseases share protein misfolding pathologies, and clinical trials have demonstrated the effectiveness of similar DBS configurations for treating both PD and AD. Additionally, ongoing research has identified fundamental selection criteria and the underlying mechanistic actions of DBS treatment in PD, providing valuable lessons to be applied in advancing the literature on using DBS for AD.

There are undoubtedly limitations to our study approach. Notably, our paper relies on a narrative search strategy, which may limit the scope of the current research literature on DBS. Despite our efforts to explore all aspects of the topic comprehensively, some literature may have been inadvertently excluded. Furthermore, it is possible that our selection criteria may have exhibited bias towards research that supports the overall narrative of our paper. However, we have taken care to ensure that our coverage of all topics is transparent and accurate, and any conflicting evidence to the overall narrative has been reported with the utmost precision.

The main goal of this study is to provide a comprehensive overview of the field of DBS, including its development, current usage, and potential future advancements. Our findings indicate that DBS systems are highly effective in treating patients with PD, where a solid understanding of the disease progression, the mechanism by which DBS affects this pathology, selection criteria for treatment, and adverse effects and their mitigations has been established. As such, the current guidelines for selecting criteria and configuring DBS systems can also be used to evaluate their efficacy in AD patients. However, further in vivo and in vitro research is necessary to ensure the precise targeting of brain regions. It is recommended to use differential HFS/LFS systems of stimulus depending on disease progression and similar age criteria for the selection of DBS as a method of best practice. Nonetheless, ethical considerations, accessibility issues, and a shortage of current research literature present ongoing challenges. Despite these obstacles, DBS is a promising tool in the treatment of neurodegenerative diseases, and establishing a solid foundation of research literature and guidelines of practice will ensure its future efficacy, reliability, and adaptability to individual patient needs.

## 7. Conclusions

Both Parkinson’s disease (PD) and Alzheimer’s disease (AD) are global health burdens characterized by neurodegeneration and amyloid protein accumulation. Due to the lack of disease-modifying pharmaceutical therapies, non-pharmaceutical therapies such as DBS have gained interest. While initial clinical trials show promise for DBS in AD, further research is required to establish its efficacy. In contrast, DBS has been used in PD for decades and is already FDA-approved. However, further clinical trials are needed to clarify the effectiveness and side effects of DBS in a variety of patients with different prognoses. This can be achieved by developing a unified system of classification with tighter controls and determining the recommended age or time after disease diagnosis that is optimal for DBS treatment. Regarding AD, foundational in vivo work to identify the most susceptible brain regions to DBS in AD-equivalent animal models is required. Clinical trials of different brain regions using either HFS or LFS systems depending on the disease prognosis of the patient should also be conducted. Continual assessment of currently identified brain regions for DBS treatment should also be conducted, as well as reviews of the current diagnostic tools and clearer criteria for selecting AD patients before DBS becomes a reliable reality for AD therapy.

## Figures and Tables

**Table 1:** A selection of conditions in which DBS is being actively researched as a viable treatment option.

Condition	Area of Stimulation	First Author	Year of Publication	References
Parkinson’s Disease	Subthalamic nucleus (STN)	Deuschl, Xie	2006, 2015	[24,25]
	Globus Pallidus internus (GPi)	Bloomstedt, Baker	2018, 2010	[26,27]
	Zona Incerta	Bloonstedt, Ossowska	2018, 2020	[26,28]
	Pedunculopontine nucleus	Thevathasan	2018	[29]
Essential Tremor	Ventral intermediate thalamus	Baizabal-Carvallo	2014	[30]
	Zona incerta	Fytagoridis	2012	[31]
Dystonia	GPi	Vidailhet	2013	[32]
Alzheimer’s Disease	Fornix			[33,34]
	Ventromedial prefrontal cortex	Mao, Lozano	2018, 2016	[35,36]
	Hippocampus	Scharre, Chakravarty	2018, 2016	[37]
	Nucleus Basalis Meynert	Kuhn	2014	[38]
Huntington’s Disease	GPi	Velez-Lago	2013	[39]
Obsessive Compulsive Disorder (OCD)	Anteromedial GPi	Nair	2014	[40]
	Nucleus Accumbens	Huff, Denys	2010, 2010	[41,42]
	Anterior limb of the internal capsule (ALIC)	Denys, Kammen	2020, 2022	[43,44]
	Ventral Capsule/Ventral Striatum (VC/VS)	Park, Kammen, Greenburg	2019, 2022, 2008	[44,45,46]
	STN	Kammen, Li, Chabardes	2022,2020, 2013	[44,47,48]
	Inferior thalamic peduncle	Germann, Lee, Kammen	2022,2019, 2022	[44,49,50]
	Bed nucleus of the stria terminalis	Luyten, Mosley, Raymaekers, Kammen	2015, 2021, 2016, 2022	[44,51,52,53]
Epilepsy	Anterior nucleus of thalamus	Salanova	2018	[54]
Depression	VC/VS	Malone	2009	[55]
	Nucleus Accumbens	Bewernick	2010	[56]

**Table 2:** Summary of all clinical trials run on DBS and AD research. The data were extracted from https://www.clinicaltrials.gov (Accessed on 19 December 2022) with 16 data points returned filtered to 14 research projects upon analysis. Abbreviations used include not disclosed (ND), year of publication (YoP), principal investigator (PI), and not published (NP).

Brain Region	Laterality	Stimulus Settings	Duration (Months)	Patients	Trial Status	YoP	Author/PI	Reference
-Fornix	Bilateral	3.9–7.5 mA, 90 µs, 130 Hz	24	1	Completed	2022	Barcia	[128]
-Fornix	ND	3.0–3.5 V, 90 µs, 130 Hz	12	6	Completed (phase I)	2010	Laxton	[37]
-Fornix	Bilateral	3.0–3.5 V, 90 µs, 130 Hz	12	42	Completed (phase II)	2016	Lozano	[34]
-Fornix	ND	ND	12	12	Active, not Recruiting	NP	Lozano	[129]
-ND	Bilateral	ND	23	3	Completed	NP	Rezai	[130]
-ND	ND	ND	12	10	Recruiting	NP	Luming	[131]
-Fornix	Bilateral	1–5 V, 90 ms, 130 Hz	12	6	Completed (phase I)	2018	Mao	[33]
-NBM	Bilateral	2.0–4.5 V, 90 µs, 20 Hz	12	6	Completed	2014	Kuhn	[38]
-NBM	ND	2.0–4.5 V, 60 µs, 20 Hz	12	30	Recruiting	NP	Chen	[132]
-NBM	Bilateral	ND	ND	6	Completed	NP	Sturm	[133]
-Hypothalamus-Fornix	Bilateral	2–3 V, 120 ms, 180 Hz	24	5	Recruiting	NP	Fontaine	[134]
-Fornix	Bilateral	ND	12	6	Completed	NP	Laxton	[135]
-Fornix	ND	ND	12	210	Recruiting	NP	ND	[136]
-Fornix-NBM	ND	ND	12	30	Recruiting	NP	ND	[137]

**Table 3:** Summary of different mechanistic pathways by which DBS affects AD and PD, as well as their consequent physiological outcomes.

Disease	Action of DBS	Physiological Effects	Year	Author	References
AD	Neuronal activation through Fornix DBS.	Increased hippocampal volume.	2020, 2012, 2019, 2015	Jakobs, Smith, Aldehri, Sankar	[142,143,144,156]
	Increased acetylcholine levels using NBM DBS.	Increased glucose uptake in amygdalo-hippocampal, temporal, and superior lingual gyrus.	2021, 2014	Maltête, Kuhn	[38,145]
	Increased prefrontal glucose uptake.	Decreased clinical decline.	2021	Lam	[146]
PD	Neuronal inhibition.	Depletion of glutamate and release of GABA and adenosine.	2012, 2001, 2001	Lozano, Contreras, Wu	[92,93,94]
	Neuronal activation.	Increased glutamate and dopamine levels.	2013, 2005, 1992	Lozano, Stefani, Benazzouz	[92,96,97]
	Neuronal activation and inhibition.	Decoupling of the soma and axons.	2013	Lozano	[92]
	Disrupt pathologic oscillatory patterns.	Neurotrophin release and generation of new neurons.	2013, 2010	Lozano, McIntyre	[92,98]

## Data Availability

Not applicable.
